# Translational maturity of artificial intelligence-enabled electrocardiography varies across cardiovascular diseases

**DOI:** 10.3389/fcvm.2026.1940621

**Published:** 2026-10-16

**Authors:** Huanhuan Chen, Jingjuan Huang, Xumin Hou

**Affiliations:** 1 Department of Biomedical Engineering, Oriental Pan-Vascular Devices Innovation College, University of Shanghai for Science and Technology, Shanghai, China; 2 Department of Cardiology, School of Medicine, Shanghai Chest Hospital, Shanghai Jiao Tong University, Shanghai, China

**Keywords:** artificial intelligence, cardiovascular disease, clinical decision support, deep learning, electrocardiography, external validation, translational maturity

## Abstract

Electrocardiography is a widely available and low-cost cardiovascular test, and artificial intelligence-enabled electrocardiography (AI-ECG) can identify latent electrical phenotypes beyond conventional interpretation. However, high retrospective discrimination does not necessarily translate into clinically reliable or actionable benefit. This critical narrative review evaluates the translational maturity of AI-ECG across arrhythmias, acute myocardial infarction, heart failure, valvular heart disease, hypertrophic cardiomyopathy, and cardiac amyloidosis. PubMed, IEEE Xplore, and Web of Science were searched from January 1, 2022, through July 10, 2026, with earlier landmark studies added by backward citation tracking. Seventy-seven publications informed the review. Evidence was appraised using five clinically oriented criteria: intended use, reference-standard reliability, external validation and transportability, clinical actionability, and effects on workflow or patient outcomes. AI-ECG has progressed furthest in ST-segment elevation myocardial infarction triage and reduced-left-ventricular-ejection-fraction screening, where prospective or randomized studies have demonstrated workflow or diagnostic-yield benefits, whereas acute myocardial infarction rule-out has reached prospective multicenter diagnostic validation. Hypertrophic cardiomyopathy screening has also advanced through international validation and prospective implementation. By contrast, occlusion myocardial infarction detection, prediction of occult or future atrial fibrillation, valvular heart disease, heart failure with preserved ejection fraction, long-term heart-failure prediction, and cardiac amyloidosis remain primarily validation-stage or risk-enrichment applications. Across domains, broader adoption is constrained by label quality, calibration, transportability, operating-threshold selection, and limited patient-outcome evidence. AI-ECG is therefore best positioned as clinician-supervised decision support linked to explicit downstream testing and care pathways.

## 1 Introduction

Cardiovascular diseases continue to be the leading cause of global mortality and represent a major challenge to healthcare systems worldwide. The global burden of cardiovascular diseases remains substantial and has increased in absolute terms alongside population aging and persistent exposure to major cardiovascular risk factors (1). Cardiovascular diseases encompass a broad spectrum of disorders affecting the heart and blood vessels, with clinical manifestations such as myocardial infarction (MI), arrhythmias, and heart failure (HF) contributing substantially to morbidity, mortality, and healthcare expenditure (2).

Electrocardiogram (ECG), which non-invasively records the electrical activity of the heart, is a fundamental, cost-effective, and ubiquitous tool in cardiovascular diagnostics. It provides critical information for identifying arrhythmias, myocardial ischemia or infarction, and electrophysiological manifestations associated with structural heart disease (3). However, the clinical utility of the ECG is constrained by several inherent limitations. Its interpretation remains dependent on clinician expertise and experience, contributing to inter-observer variability, particularly when abnormalities are subtle or recordings are affected by noise (4, 5). The growing volume of long-term monitoring data generated by Holter monitors and wearable devices has made manual beat-by-beat analysis increasingly labor-intensive and inefficient (6). More importantly, conventional ECG interpretation may miss occult or subclinical disease. Conditions such as paroxysmal atrial fibrillation (PAF) and asymptomatic left ventricular systolic dysfunction may be associated with subtle electrophysiological signatures that are not consistently recognized through conventional visual interpretation (7). These limitations have motivated the development of computational approaches that can extract latent ECG phenotypes beyond conventional waveform measurements.

Earlier automated ECG systems relied mainly on traditional machine-learning methods, including support vector machines, k-nearest neighbors, and decision trees, using predefined features such as QRS duration, ST-segment deviation, and RR-interval variability (8). Although clinically interpretable, these features may not capture nonlinear temporal dynamics, subtle morphological variation, or distributed inter-lead relationships. Deep learning (DL) has therefore enabled end-to-end representation learning directly from raw waveforms, ECG images, and multi-lead spatial representations.

The promise of artificial intelligence-enabled electrocardiography (AI-ECG), particularly DL-based approaches, extends beyond automating established diagnostic rules by identifying latent electrophysiological patterns that may not be apparent on conventional interpretation (9). For example, DL has identified hypertrophic cardiomyopathy from ECG images, with discriminative regions involving clinically relevant precordial and lateral leads (10), while an ECG-based DL score has provided prognostic stratification after cardiac resynchronization therapy beyond guideline criteria evaluated in that study (11). These findings suggest that routine ECGs may contain clinically relevant information beyond visually apparent abnormalities. AI-ECG may therefore complement conventional interpretation in diagnostic assessment, opportunistic screening, and risk stratification, while remaining linked to clinician review and confirmatory evaluation rather than autonomous diagnosis (Figure 1).

**Figure 1:**
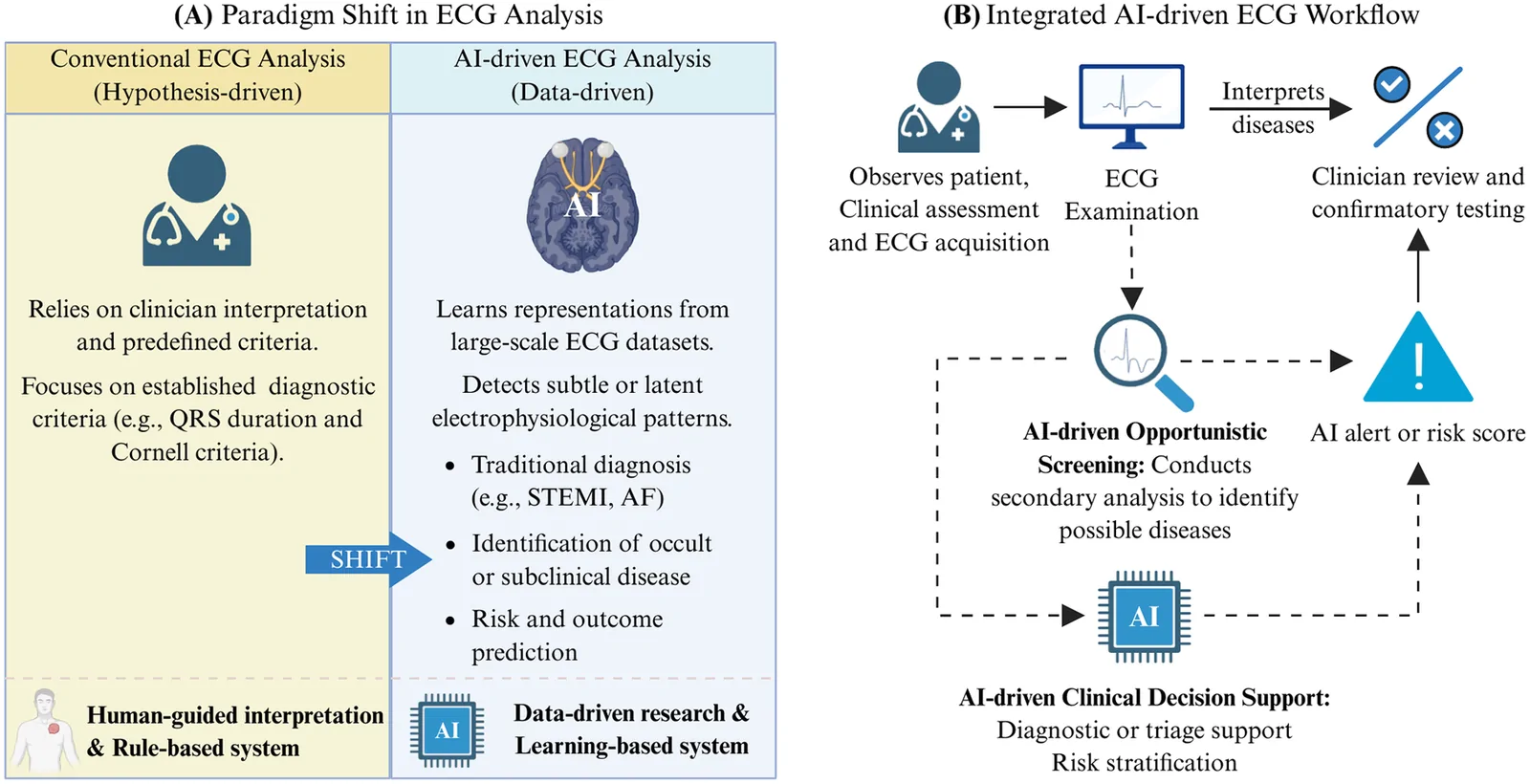
Paradigm shift from conventional to AI-enhanced ECG interpretation and its integration into clinical workflows. **(A)** AI-ECG extends rule-based interpretation by identifying subtle electrophysiological patterns, occult disease, and future risk. **(B)** Within clinical workflows, AI supports opportunistic screening, diagnostic or triage assessment, and risk stratification, while final decisions remain subject to clinician review and confirmatory testing. Created in BioRender. Chen, H. (2026) https://BioRender.com/ue8s9ug

However, high retrospective discrimination should not be equated with clinical utility. AI-ECG models may capture disease-related electrophysiological patterns but may also exploit shortcuts associated with demographics, comorbidities, acquisition devices, filtering, institutional practice, or label-generation processes. Accordingly, an increasingly important question is not only whether AI-ECG models achieve high AUROC values, but whether their predictions are valid, generalizable, calibrated, interpretable, and clinically actionable (7, 9).

Recent reviews have synthesized AI-ECG architectures, disease applications, and implementation barriers, including bench-to-bedside and regulatory considerations (7, 9). These reviews are commonly organized around model type, disease category, or broad implementation themes. The present review instead compares heterogeneous cardiovascular applications using a common set of intended-use-specific translational criteria. Diagnostic classification, opportunistic screening, risk prediction, emergency triage, and longitudinal monitoring differ in their target populations, reference standards, acceptable error costs, operating thresholds, and downstream clinical actions. Reported discrimination should therefore be interpreted together with label reliability, patient-level data separation, external validation, calibration, clinically relevant predictive values, and evidence that model outputs change subsequent care.

The distinctive contribution of this review is a cross-application comparison of translational maturity using a common set of clinically oriented criteria, rather than a catalogue of model architectures or reported discrimination alone. Specifically, we evaluate whether performance evidence is supported by a clinically appropriate intended use, a credible reference standard, external validation and transportability, actionable model outputs, and evidence that the model influences clinical workflow or patient outcomes. This framework enables direct comparison of heterogeneous AI-ECG applications at different stages of translation and helps distinguish technically promising models from applications supported by evidence for real-world clinical use.

We therefore apply this unified framework across cardiovascular applications from an algorithm-to-clinical-application perspective. We examine how ECG data foundations, preprocessing, task-oriented representation learning, validation design, and workflow integration shape the credibility and clinical relevance of the evidence. We critically appraise AI-ECG applications in arrhythmia classification, acute myocardial infarction and occlusion-oriented reperfusion triage, heart failure screening and risk stratification, valvular heart disease assessment, and opportunistic screening for hypertrophic cardiomyopathy and cardiac amyloidosis. Across these domains, we ask five translational questions:

1. What is the intended clinical use of the model?
2. How reliable is the reference standard or disease label?
3. Has the model been externally validated across populations, institutions, devices, and acquisition settings?
4. Are the predictions interpretable and clinically actionable?
5. Is there evidence that the model improves workflow efficiency, diagnostic decision-making, or patient outcomes?

Applying this framework, we compare whether the evidence for each application remains at the stage of retrospective technical feasibility or has progressed to external validation, prospective implementation, workflow evaluation, or patient-outcome assessment. This structured comparison distinguishes technical performance from translational maturity, identifies applications with comparatively stronger clinical evidence, and clarifies the methodological gaps that remain before broader implementation. Sections 2–4 describe the literature-selection strategy and the physiological and engineering foundations relevant to evidence reliability. Section 5 applies the framework across major cardiovascular applications, and Section 6 compares their translational maturity and outlines priorities for validation, implementation, governance, and future research.

## 2 Literature search and evidence selection

This article was designed as a clinically oriented critical narrative review. Its purpose was to compare how translational maturity varies across AI-ECG applications, rather than to provide an exhaustive study-level synthesis of a single narrowly defined clinical question. The review integrates technical, diagnostic, implementation, workflow, and governance evidence across applications that differ in intended use, target population, ECG format, reference standard, validation strategy, operating threshold, downstream clinical action, and reported endpoint. A critical narrative design allowed these distinct forms of evidence to be interpreted within their clinical contexts and compared using a common translational framework. A structured multi-database search and predefined appraisal dimensions were retained to improve transparency and consistency. Because no formal study-level risk-of-bias assessment was performed, the conclusions should be interpreted as a structured narrative appraisal rather than a systematic evidence synthesis or validated quantitative ranking.

### 2.1 Search scope and evidence identification

PubMed, IEEE Xplore, and Web of Science were searched for publications from January 1, 2022, through July 10, 2026. The 2022 lower boundary was selected as a pragmatic starting point for the structured search to emphasize the contemporary translational phase of AI-ECG research, in which evidence increasingly extended beyond retrospective model development toward independent validation, prospective implementation, and workflow-oriented evaluation. It was not intended as an exclusion boundary for historically important evidence. Earlier seminal studies were therefore retained through backward citation tracking when they established the origins of major AI-ECG applications, introduced pivotal models, or provided clinically important validation evidence necessary for interpreting subsequent translational progress. Database-specific syntax was adapted to the indexing terms and searchable fields available in each source. The search combined three major concept blocks: artificial intelligence methods, ECG data, and cardiovascular conditions or clinical applications. AI-related terms included “artificial intelligence,” “machine learning,” “deep learning,” “convolutional neural network,” “recurrent neural network,” “transformer,” “graph neural network,” “representation learning,” “multimodal learning,” and “explainable artificial intelligence.” ECG-related terms included “electrocardiogram,” “electrocardiography,” “ECG,” “12-lead ECG,” “ambulatory ECG,” “Holter,” and “wearable ECG.” Clinical and application-related terms included arrhythmia, atrial fibrillation, myocardial infarction, occlusion myocardial infarction, heart failure, left ventricular dysfunction, valvular heart disease, hypertrophic cardiomyopathy, cardiac amyloidosis, screening, diagnosis, risk prediction, triage, external validation, clinical implementation, workflow, and patient outcomes. Terms within each block were combined with OR, and the concept blocks were combined with AND.

Reference lists of relevant reviews and key original studies were also examined to identify additional relevant publications that might not have been retrieved consistently because of database-indexing differences. Publications identified through backward citation tracking were considered according to the same selection and prioritization principles described in Section 2.2. Foundational and landmark publications predating the predefined search period were included selectively when necessary to establish historical context, explain ECG physiology or methodological principles, describe the origins of clinically important AI-ECG applications, or provide pivotal validation evidence. This supplementary approach accounts for the inclusion of earlier publications concerning ECG physiology, conventional machine learning, atrial fibrillation prediction, myocardial infarction localization, ventricular dysfunction screening, VHD, and hypertrophic cardiomyopathy.

### 2.2 Evidence selection and narrative appraisal

Records were first assessed by title and abstract, followed by full-text evaluation when relevant. Screening was conducted by HC; independent duplicate screening was not performed. The organization of the selected evidence and its clinical interpretation were subsequently reviewed by the coauthors. Peer-reviewed original studies were selected when they applied artificial intelligence, machine learning, or deep learning to ECG signals, ECG images, ambulatory recordings, wearable ECGs, or ECG-derived representations and addressed a clinically relevant detection, classification, screening, diagnostic-triage, prognostic, or decision-support task. Both ECG-only and multimodal studies were considered when ECG data constituted a substantive model input. Multimodal studies were interpreted as evidence for integrated decision-support systems rather than standalone AI-ECG unless ECG-only performance was reported separately. Technical studies were included selectively when they clarified preprocessing, segmentation, ECG representation, inter-lead modeling, multimodal integration, federated learning, or interpretability issues with direct implications for physiological fidelity, generalizability, or clinical reliability. Reviews, clinical guidelines, technical standards, and regulatory documents were used for background, definitions, methodological context, interoperability, and governance, but were not treated as primary clinical-performance evidence.

Studies were prioritized for detailed appraisal when they contributed evidence regarding intended clinical use, reference-standard reliability, external validation and transportability, clinical actionability, or effects on workflow, clinical decisions, healthcare utilization, or patient outcomes. Particular attention was given to studies with patient-level data separation, independent or international validation, multicenter evaluation, prospective implementation, controlled workflow assessment, clinically relevant operating thresholds, or patient-level outcome measures. When several studies addressed the same application, reports providing more informative validation or implementation evidence were emphasized, while distinct external-validation or prospective studies were retained. Overall, 77 publications informed the final synthesis. Forty representative AI-ECG application studies are summarized in Tables 1–6, while additional publications were used to support physiological, methodological, guideline, standardization, and governance considerations.

**Table 1:** Summary of related studies on DL for arrhythmia detection, prediction, and classification.

Reference	Clinical Task	Model Inputs	Model Type	Results
Attia et al., Lancet, 2019 (27)	Identification of AF from sinus-rhythm ECG	Standard 10-s 12-lead ECG, 500 Hz; 8 independent leads used	CNN with residual blocks	AUC 0.87 single ECG; AUC 0.90 multiple ECGs
Yuan et al., JAMA Cardiology, 2023 (28)	Prediction of future AF from sinus-rhythm ECG	12-lead ECG (10-second, 250 Hz)	CNN	AUROC: 0.93 Accuracy: 0.87 Sensitivity: 0.87 Specificity: 0.87
Andayeshgar et al., IJERPH, 2022 (29)	Arrhythmia diagnosis	12-lead ECG (10-second, 500 Hz)	GCN with mutual information adjacency matrix	Accuracy: 99.71% Sensitivity: 98.45% Specificity: 99.85% Precision: 97.89%
Yaser et al., Computers in Biology and Medicine, 2025 (30)	Cardiac arrhythmia detection	Lead II used for graph construction (10-second recordings, 500 Hz)	TCN feature extractor with spectral GCN on cycle graphs	Accuracy: 95.5%
Jiang et al., Computing in Cardiology, 2020 (31)	Multi-label cardiac disorder diagnosis	12-lead ECG (18-second segments, resampled to 500 Hz)	ResNet-Bi-GRU feature extractor with GCN for label dependency modeling	Challenge Score: 0.627 F1 Score: 0.603
Li et al., Health Information Science and Systems, 2022 (32)	Multi-label ECG anomaly classification	12-lead ECG (10-second segments, resampled to 500 Hz)	Bi-LSTM on binary relevance^b^	F1 Score: 0.767 Hamming Loss: 0.073 Coverage: 3.40 Ranking Loss: 0.262
Liu et al., Biosensors, 2021 (33)	Multi-label ECG classification	12-lead ECG (20-second segments, resampled to 250 Hz)	1D ResNet with class-wise attention	Cost-Weighted Accuracy: 0.641 ± 0.009
Yang et al., IEEE Trans. Emerg. Topics Comput. Intell., 2023 (34)	Multi-label ECG classification	12-lead ECG (10-second recordings, 100 Hz)	Multi-view multi-scale CNN with coordinate attention and knowledge distillation	Macro-AUC: 92.89% (all tasks), 94.35% (diagnostic), 95.65% (rhythm) on PTB-XL^a^

Bi-GRU, bidirectional gated recurrent unit; Bi-LSTM, bidirectional long short-term memory; TCN, temporal convolutional network.

a PTB-XL is a large publicly available clinical electrocardiography dataset.

b Binary relevance is a problem transformation method for multi-label classification.

**Table 2:** Summary of related studies on DL for AMI detection, localization, and occlusion-oriented triage.

Reference	Clinical Task	Model Inputs	Model Type	Results
Chen et al., Biosensors, 2025 (18)	MI detection and localization	12-lead ECG heartbeat + Lead II frequency-domain spectral image	MFF-CNN: 1D CNN + two 2D CNNs	Inter-patient validation: detection accuracy 99.98%; localization accuracy 84.86%
Chen et al., Front. Cardiovasc. Med., 2022 (36)	Prehospital STEMI detection	12-lead ECG (10-second recordings, 500 Hz)	CNN-LSTM	Accuracy: 99.2% Sensitivity: 94.1% Specificity: 99.4% Precision: 88.9% F1: 0.914 AUC: 0.997
Lin et al., NEJM AI, 2024 (37)	Rapid identification of STEMI	12-lead ECG (10-second recordings, 500 Hz)	CNN-LSTM-based STEMI detection algorithm	PPV: 89.5% (95% CI, 85.3–93.6%) NPV: 99.9% (95% CI, 99.9–100.0%) Sensitivity: 89.5% Specificity: 99.9%; AI-assisted triage reduced door-to-balloon and ECG-to-balloon times
Lee et al., European Heart Journal, 2025 (38)	Rule-out AMI in emergency department	12-lead ECG (10-second recordings, 500 Hz)	ResNet-based AI-ECG (AiTiAMI)	AUROC: 0.878 (AMI) low-risk threshold (score < 3.0): sensitivity 99.6% NPV 99.1%
Xiong et al., Comput. Methods Programs Biomed., 2021 (39)	Myocardial infarction localization (11 types)	12-lead ECG (heartbeats segmented around R-peak, 1000 Hz)	DenseNet-based multi-lead ECG classifier	Accuracy: 99.87% Sensitivity: 99.84% Specificity: 99.98%
Kim et al., Frontiers in Physiology, 2024 (40)	STEMI detection and infarction territory classification	12-lead ECG images (scanned paper ECGs, 1228 × 2460 resolution)	Faster R-CNN for ST-segment elevation detection with weighted ensemble classifier	STEMI: AUROC 0.939, Accuracy 89.5%; Anterior MI: AUROC 0.955, Accuracy 91.9%; Inferior MI: AUROC 0.966, Accuracy 95.4%; Lateral MI: AUROC 0.925, Accuracy 89.8%
Herman et al., Eur. Heart J. Digit. Health, 2024 (42)	OMI detection for acute coronary occlusion	12-lead ECG (10-second recordings, 500 Hz)	Deep CNN with lead-specific feature extraction	AUC: 0.938 (95% CI, 0.924–0.951) Accuracy: 90.9% Sensitivity: 80.6% Specificity: 93.7% PPV: 78.0% NPV: 94.6%
Riek et al., IEEE Trans. Biomed. Eng., 2025 (43)	OMI detection	12-lead ECG median beats (400 datapoints per lead, 250 Hz)	ECG-SMART-NET: ResNet-18 modified with temporal (1 × k) followed by spatial (12 × 1) convolution	AUC: 0.953 (95% CI, 0.921–0.978) Average Precision: 0.843 F1: 0.785 Sensitivity: 67.7% Specificity: 99.6% Accuracy: 96.9%

MFF-CNN, multi-domain feature fusion model comprising one 1D CNN and two 2D CNNs.

**Table 3:** Summary of related studies on DL for heart failure screening and risk stratification.

Reference	Clinical Task	Model Inputs	Model Type	Results
Li et al., Biomed Signal Process Control, 2019 (45)	Automatic staging of heart failure (5 classes: N, A, B, C, D)	Lead II ECG segments combined with reported clinical features	CNN-RNN (CNN feature extractor and LSTM classifier)	Accuracy: 97.6% Sensitivity: 96.3% Specificity: 97.4%
Attia et al., Int. J. Cardiol., 2021 (46)	Detection of left ventricular systolic dysfunction (LVEF ≤ 35%)	12-lead ECG (10-second recordings, 500 Hz)	CNN	AUC: 0.82 Accuracy: 97.0% Sensitivity: 26.9% Specificity: 97.4%
Hou et al., Can. J. Cardiol., 2025 (47)	Detection of low left ventricular ejection fraction (LVEF ≤ 50%)	12-lead ECG images (2.5-second waveforms, extracted from PDF, 500 Hz)	DenseNet-121-based CNN	AUC: 0.965 Accuracy: 92.8% Sensitivity: 88.8% Specificity: 92.9%
Choi et al., Sci. Rep., 2022 (48)	HFrEF identification in acute symptomatic HF	12-lead ECG (10-second recordings, 500 Hz)	CNN-based algorithm (DeepECG-HFrEF)	AUC: 0.844 Sensitivity: 77.9% Specificity: 76.3% Accuracy: 77.0% PPV: 70.8% NPV: 82.4%
Bachtiger et al., Lancet Digit. Health, 2022 (49)	Point-of-care screening for LVEF ≤40%	Single-lead ECG (15-second recordings, 10-second analyzed) via ECG-enabled stethoscope at 5 anatomical positions	CNN	AUC: 0.91 Sensitivity: 91.9% Specificity: 80.2%
Hong et al., Eur. Heart J. Digit. Health, 2025 (50)	HFpEF identification using HFA-PEFF ≥5	12-lead ECG (10-second recordings, 500 Hz)	1D DenseNet-121 based CNN	AUROC: 0.81 (95% CI, 0.79–0.82); HR: 9.56 for cardiac death and 5.91 for HF hospitalization in the AI-ECG-positive group
Chen et al., IEEE Trans. Big Data, 2025 (23)	HF risk prediction in UKB-HYP and UKB-MI cohorts	12-lead ECG with ECG-report alignment pretraining	LLM-informed ECG dual-attention network	C-index: 0.6349 in UKB-HYP; 0.5805 in UKB-MI
Park et al., Korean J. Intern. Med., 2026 (24)	Detection of HFrEF	8-lead ECG (I, II, V1–V6) and clinical metadata (age, NT-proBNP, sodium)	EfficientNet B3-based CNN	AUC: 0.95 Sensitivity: 54.8% Specificity: 96.9% PPV: 61.0% NPV: 95.2%
Tsai et al., BMC Medicine, 2025 (51)	AI-ECG decision support for reduced LVEF detection in non-cardiology inpatients	12-lead ECG integrated into hospital information system	Pragmatic RCT using AI-ECG alert	Higher 30-day diagnosis of reduced LVEF, echocardiography PPV (34.2% vs. 20.2%), and cardiology consultation (29.3% vs. 23.5%), without greater echocardiography use

HF, heart failure; HFrEF, heart failure with reduced ejection fraction; HFpEF, heart failure with preserved ejection fraction; LVEF, left ventricular ejection fraction; RNN, recurrent neural network; LLM, large language model; AUC, area under the curve; CI, confidence interval; PPV, positive predictive value; NPV, negative predictive value; HR, hazard ratio; RCT, randomized controlled trial.

**Table 4:** Summary of related studies on DL for valvular heart disease.

Reference	Clinical Task	Model Inputs	Model Type	Results
Elias et al., JACC, 2022 (52)	Detection of left-sided VHDs (AS, AR, MR)	12-lead ECG (10-second recordings, 250 Hz) and demographic and ECG-specific tabular data (age, PR interval, etc.)	ValveNet (1D-CNN)	AUROC: 0.88 (AS), 0.77 (AR), 0.83 (MR) Sensitivity: 78% Specificity: 73% PPV: 20% NPV: 97.6%
Kwon et al., Journal of Electrocardiology, 2020 (53)	Detection of moderate-to-severe mitral regurgitation	12-lead ECG (8-second recordings, 500 Hz)	CNN with residual blocks	12-lead AUROC: 0.816 internal, 0.877 external; single-lead AUROC: 0.758 internal, 0.850 external
Lin et al., AGING, 2024 (54)	Screening for multiple VHDs (AS, AR, PR, TR, MR)	12-lead ECG (10-second recordings, 500 Hz) with clinical metadata (age, sex)	CNN	AUC: 0.874 (AS), 0.802 (AR), 0.793 (PR), 0.841 (TR), 0.828 (MR)
Liang et al., European Heart Journal, 2025 (55)	Diagnosis and prediction of future significant regurgitant VHDs (MR, AR, TR)	12-lead ECG (10-second recordings, 400 Hz)	CNN-based survival neural network	Diagnosis (external AUROC): 0.777 (MR), 0.751 (AR), 0.805 (TR); Prediction (external C-index): 0.698 (MR), 0.647 (AR), 0.702 (TR)
Nagai et al., Frontiers in Radiology, 2025 (56)	Early detection of AS	12-lead ECG (2-second segments, 500 Hz) and chest x-ray images	1D ResNet50-Transformer (ECG) and EfficientNet (CXR)	AUROC: 0.812 (multimodal), 0.775 (ECG-only), 0.755 (CXR-only)

VHD, valvular heart disease; AS, aortic stenosis; AR, aortic regurgitation; MR, mitral regurgitation; TR, tricuspid regurgitation; PR, pulmonary regurgitation; PPV, positive predictive value; NPV, negative predictive value; CXR, chest x-ray.

**Table 5:** Summary of related studies on DL for hypertrophic cardiomyopathy.

Reference	Clinical Task	Model Inputs	Model Type	Results
Sangha et al., Nature Cardiovascular Research, 2025 (10)	HCM detection from ECG images	12-lead ECG images; imaging-based labels in development and external cohorts, diagnosis-code labels in UKB	EfficientNet-B3-based CNN with self-supervised pretraining; Grad-CAM	AUROC: 0.95 internal testing, 0.94 MIMIC-IV, 0.92 AUMC, 0.91 UKB; key regions localized to V4–V5
Ko et al., JACC, 2020 (57)	HCM detection	Standard 10-s 12-lead ECG; verified HCM cases from a dedicated HCM clinic; age- and sex-matched controls	CNN	AUC: 0.96; sensitivity: 87%; specificity: 90%; AUC 0.95 in ECG-LVH and normal-ECG subgroups
Siontis et al., European Heart Journal—Digital Health, 2024 (58)	International external validation of HCM detection	Raw 10-s 12-lead ECGs; HCM defined by ESC/ACC/AHA criteria using echocardiography or CMR; cohorts from Switzerland, UK, and South Korea	CNN-based Mayo Clinic AI-ECG algorithm	AUC: 0.922; accuracy: 86.9%; sensitivity: 82.8%; specificity: 87.7%; age/sex-matched AUC: 0.921
Goto et al., Circulation, 2022 (59)	HCM detection and differentiation from LVH mimics	12-lead ECGs and echocardiograms from academic centers in the USA and Japan	Federated learning-based ECG and echocardiography models	C-statistics: 0.90–0.96 for ECG and 0.90–0.96 for echocardiography; ECG model differentiated HCM from hypertension, AS, and CA with C-statistics of 0.84, 0.83, and 0.88
Love et al., Heart, 2025 (60)	Prospective clinical implementation of AI-ECG alert for suspected HCM	Algorithm-compliant 12-lead ECGs from five healthcare systems; adults without prior HCM diagnosis	FDA-cleared software alert system, Viz HCM	145,848 patients screened; 4,348 alerts; 69% alert view rate; 182/217 enrolled patients had follow-up actions; 17 new HCM diagnoses; median ECG-to-diagnostic imaging time: 7.5 days

ACC/AHA, American college of cardiology/American heart association; AUMC, Amsterdam university medical center; ESC, european society of cardiology; FDA, U.S. food and drug administration; Grad-CAM, gradient-weighted class activation mapping; MIMIC-IV, medical information mart for intensive care IV; UKB, UK biobank.

**Table 6:** Summary of AI-based approaches for cardiac amyloidosis screening and differential diagnosis.

Reference	Clinical Task	Model Inputs	Model Type	Results
Schrutka et al., Heart, 2022 (61)	CA detection	ECGI and 12-lead ECG; ATTR-CA cases with HFpEF/HCM disease controls	ECGI-derived ML algorithm translated into visual 12-lead ECG rule	Cardiologist AUC from 0.69 to 0.97; sensitivity 0.32→0.89; specificity 0.80→0.96
Haimovich et al., Cardiovascular Digital Health Journal, 2023 (62)	Classification of LVH etiologies, including CA	10-s 12-lead ECGs	PCLR-based ECG representation with logistic regression; 12-lead and lead I/II LVH-Net models	CA AUROC 0.95 with 12-lead LVH-Net; single-lead models retained favorable performance
Harmon et al., JACC: Advances, 2023 (63)	Postdevelopment validation of AI-ECG for CA detection	10-s 12-lead ECGs; AL confirmed by biopsy and ATTR by biopsy or positive 99mTc-PYP scintigraphy; 440 CA cases and 6,600 matched controls	CNN-based Mayo Clinic AI-ECG model	AUC: 0.84; sensitivity: 64.3%; specificity: 90.4%. Lower performance in Hispanic patients, LVH, and LBBB; internal temporal validation only
González-López et al., Heart Rhythm, 2026 (64)	ATTR-CM detection and ECG-based triage	12-lead ECGs from patients evaluated for ATTR-CM; ATTR-CM diagnosed by invasive or ESC-consistent noninvasive criteria	Willem AI platform; time-slicing CNN with max-pooling	AUC: 0.88; sensitivity: 80.7%; specificity: 78.5%; sensitivity decreased to 68.4% in asymptomatic patients
Zhang et al., BMC Medicine, 2026 (25)	Multimodal differential diagnosis of CA, HCM, and HHD	ECG, echocardiographic, and clinical variables; CA mainly biopsy-confirmed	Seven-variable Super Learner with SHAP interpretation and simplified score	External AUC: 0.96 for CA, 0.93 for HCM, 0.91 for HHD. WeChat-based tool and three-tier triage proposed; simplified score sensitivity 75.4%

ATTR-CA, transthyretin cardiac amyloidosis; ECGI, electrocardiographic imaging; LBBB, left bundle branch block; ML, machine learning; PCLR, patient contrastive learning of representations; SHAP, shapley additive explanations; 99mTc-PYP, technetium-99 m pyrophosphate; HHD, hypertensive heart disease.

Because the included studies differed substantially in clinical tasks, populations, ECG formats, reference standards, validation strategies, disease prevalence, operating thresholds, and reported outcomes, evidence was synthesized narratively rather than quantitatively. No quantitative meta-analysis or formal risk-of-bias assessment was performed. A formal study-level risk-of-bias or reporting-quality assessment was not prespecified because this critical narrative review was designed to compare translational evidence across heterogeneous study types rather than to estimate pooled effects or formal certainty of evidence. This methodological choice is acknowledged as a limitation of the review. For each representative application study, appraisal was conducted using the five clinically oriented criteria defined in the Introduction: intended clinical use, reference-standard reliability, external validation and transportability, clinical actionability, and evidence of effects on workflow or patient outcomes. Translational maturity was interpreted according to the strongest evidence type identified for each application, including patient-level model development, independent transportability, prospective diagnostic validation or implementation, controlled workflow evaluation, and patient-outcome assessment. These stages were used to distinguish retrospective technical performance from evidence of clinical utility and should be interpreted as a review-specific narrative framework rather than a validated quantitative grading system or formal ranking of individual models. The five appraisal criteria were not numerically weighted or combined into a maturity score. They were used as a structured interpretive framework because no validated weighting or threshold system currently supports quantitative ranking across clinically heterogeneous AI-ECG applications. Accordingly, cross-application comparisons should be interpreted as qualitative evidence-based judgments rather than statistically demonstrated differences between disease domains.

## 3 ECG data foundations, segmentation, and temporal standardization

### 3.1 Physiological and spatial foundations of ECG

ECG data sources include resting ECG, ambulatory ECG, and exercise stress testing, all of which rely on the principle that the human body acts as a volume conductor, transmitting electrical potentials generated by cardiac excitation to the body surface (12). At a fundamental level, the ECG records ionic currents generated by transmembrane ion fluxes in myocardial cells. Electrical impulses originate from the sinoatrial node and propagate through the atrioventricular node and the His–Purkinje system, coordinating atrial and ventricular contractions to maintain effective systemic perfusion. During this process, bioelectric currents generated by myocardial depolarization and repolarization propagate through body tissues to the surface, forming spatially distributed potential gradients. By placing electrodes at specific anatomical locations, ECG systems detect potential differences and record continuous cardiac electrical activity. ECG is a noninvasive and cost-effective diagnostic modality routinely used in clinical practice to assess cardiac electrophysiological abnormalities. A typical cardiac cycle consists of the *P* wave (atrial depolarization), the QRS complex (ventricular depolarization), and the T wave (ventricular repolarization). The morphology, amplitude, and temporal intervals of these waveforms reflect pathological states of cardiac function (13).

Standard clinical ECG monitoring uses the 12-lead system (I, II, III, aVR, aVL, aVF, V1–V6) to provide a spatial representation of cardiac electrical patterns. Einthoven's law states that any two independent limb leads are sufficient to derive the remaining limb lead using linear relations (14). Furthermore, Frank's vectorcardiographic framework represents cardiac electrical activity as an equivalent dipole in three-dimensional space (15). Surface ECG leads record the projections of this dipole along specific axes. Consequently, the standard 12-lead ECG should not be viewed as 12 mutually independent time series, but rather projections of a common cardiac electrical source onto different spatial planes, including the frontal and horizontal planes. This multi-lead spatial projection information is used to diagnose patients and enables precise localization of infarcted territories through established lead-region correspondence. For example, inferior MI is typically associated with ST-segment changes in leads II, III, and aVF. The morphological components and spatial organization of the standard 12-lead ECG provide the physiological basis for AI-based ECG representation learning (Figure 2).

**Figure 2:**
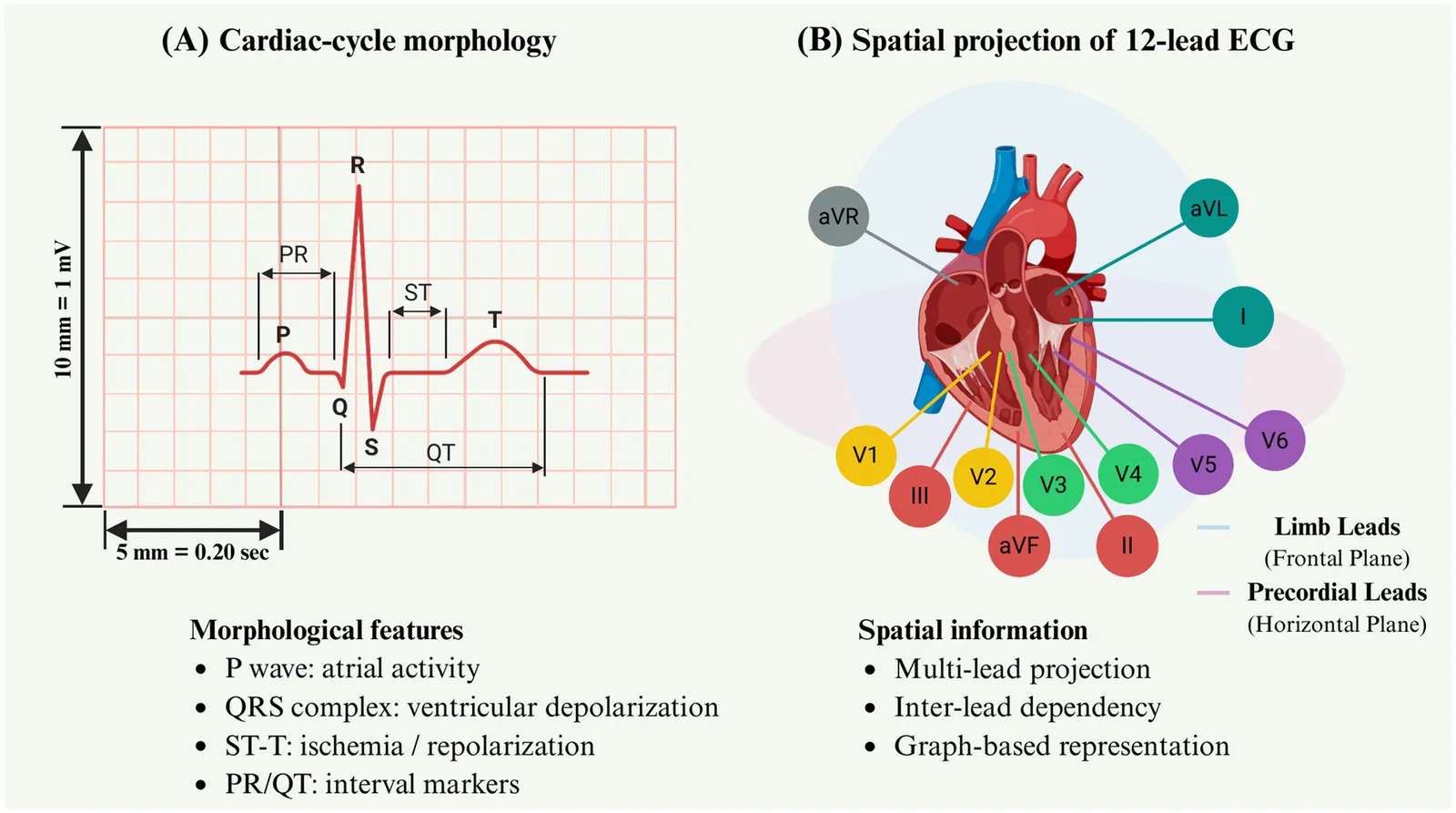
Morphological and spatial foundations of ECG representation for AI-based analysis. **(A)** A standard cardiac cycle contains *P* wave, QRS complex, T wave, and clinically relevant intervals or segments, including PR interval, ST segment, and QT interval. These waveform components provide morphological and interval-based information for AI-ECG models. **(B)** The standard 12-lead ECG captures cardiac electrical activity from complementary spatial projections. Limb leads primarily reflect the frontal plane, whereas precordial leads reflect the horizontal plane. This spatial organization provides the physiological basis for multi-lead representation learning and inter-lead dependency modeling. Created in BioRender. Chen, H. (2026) https://BioRender.com/9t1gp0p

### 3.2 ECG segmentation and temporal alignment

The choice between window-level and beat-level segmentation depends on the intended clinical task. In the former, long ECG recordings are divided into fixed-duration frames, such as 10- or 30-second windows. Multi-lead ECG recordings are represented as C × T tensors, where C denotes the number of leads and T denotes the temporal length. This strategy preserves continuous rhythm information, making it suitable for tasks involving temporal irregularity, long-term risk prediction and capturing rhythm dynamics over multiple cardiac cycles. Examples include atrial fibrillation (AF) and Holter-based event detection. In contrast, beat-level approaches commonly use an R-peak detector, such as the Pan–Tompkins algorithm, to extract fixed temporal windows around individual heartbeats (16). This approach is suitable for tasks in which local morphology serves as the primary diagnostic basis. For example, beat-level representations could be useful for tasks such as premature ventricular contraction classification and ST-segment deviation assessment in MI. Therefore, the ECG segmentation strategy should be selected with care based on the underlying electrophysiological substrate of the target disease.

However, most beat-level segmentation focuses on the shape of individual heartbeats, ignoring the temporal dependence between adjacent beats and the dynamic variation of RR intervals. To address this issue, Liu et al. (17) extracted nine temporal and spatial features, including anterior and posterior RR intervals and their ratio, as well as the skewness and kurtosis of the current heartbeat. These features were then concatenated with deep representations learned by a multi-layer network. This strategy mimics clinical practice, which involves jointly assessing waveform morphology and rhythm patterns. It also improves classification performance for supraventricular ectopic beats.

Reliable extraction of both morphological and rhythm features critically depends on the physical fidelity of the input heartbeat segments. However, sampling frequencies vary across different datasets. Using a fixed number of sampling points for heartbeat segmentation results in inconsistent temporal durations, leading to time scaling and morphological distortion of the electrocardiogram waveforms. This reduces the consistency of feature representation and adversely affects model learning. To address this issue, Liu et al. (17) proposed the principle of ‘physical time consistency’. For datasets with different sampling frequencies, such as SVDB and INCART, heartbeat segments were extracted using a fixed temporal window (0.25 s before and 0.44 s after the R peak), followed by resampling to ensure consistent input length. Similarly, Chen et al. (18) resampled ECG signals from different datasets to a common sampling frequency before performing heartbeat segmentation using a fixed number of sampling points (250 samples before and 400 samples after the R peak). This strategy reduces artifacts introduced by heterogeneous sampling rates and may encourage models to learn more physiologically meaningful morphology.

## 4 Task-oriented representation learning in AI-ECG

### 4.1 Handcrafted ECG features as interpretable baselines

Although DL has become the predominant approach in AI-ECG, conventional machine-learning methods remain useful as interpretable baselines when the target condition is associated with established ECG measurements. Features such as waveform amplitudes and durations, electrical axes, intervals, and ST–T deviations are directly linked to recognized electrophysiological concepts, allowing clinicians to examine the physiological basis of a prediction. For example, a random-forest model using conventional ECG descriptors improved the identification of non-ST-segment elevation acute coronary syndrome compared with interpretation based mainly on ST-segment changes (19). Such findings suggest that combinations of established ECG features may provide information beyond individual diagnostic criteria. Nevertheless, interpretability alone does not establish clinical utility; these models must still demonstrate incremental value within a defined diagnostic or screening pathway.

### 4.2 Morphological, temporal, spatial, and multimodal ECG representations

Deep-learning models extend handcrafted analysis by extracting subtler representations directly from ECG signals. Morphology-oriented models, most commonly based on convolutional neural networks (CNNs), capture local waveform patterns involving the *P* wave, QRS complex, and ST–T segments. These representations can be linked to different clinical substrates: *P*-wave abnormalities may reflect atrial remodeling and support AF risk enrichment; ST–T changes may indicate myocardial ischemia relevant to acute myocardial infarction (AMI) or occlusion myocardial infarction (OMI); and QRS or repolarization abnormalities may reflect conduction disturbance or myocardial remodeling associated with left ventricular dysfunction, HF, valvular heart disease (VHD), HCM, and cardiac amyloidosis. Because these signatures are not necessarily disease-specific, model outputs should generally support screening or diagnostic triage rather than autonomous diagnosis.

Temporal representations are important when clinically relevant information depends on beat-to-beat variation or longer rhythm context. Recurrent models, including long short-term memory (LSTM) networks, as well as temporal convolutional networks and Transformers, can capture RR-interval dynamics, transient rhythm transitions, and event boundaries. These properties are particularly relevant to paroxysmal AF detection, Holter review, and wearable monitoring, in which isolated-beat classification may miss intermittent or evolving abnormalities. Sequence-aware models have, for example, been used to detect AF and identify episode onset and offset in dynamic ECG recordings (20).

Spatial representation learning addresses the complementary information contained across ECG leads. Although conventional multi-lead models can learn cross-lead associations, they do not necessarily encode the physiological organization of the 12-lead system explicitly (21). Graph-based and lead-attention approaches can represent relationships among leads and may support infarct localization, conduction-abnormality recognition, and the identification of spatially distributed patterns associated with structural heart disease (22). However, learned or predefined inter-lead relationships should not automatically be interpreted as anatomical mechanisms, and current evidence does not establish that graph neural networks consistently outperform simpler multi-lead models in external clinical validation.

Emerging multimodal models extend ECG representation learning by integrating ECG waveforms with imaging, clinical reports, electronic health records, biomarkers, or longitudinal outcomes, sometimes through cross-modal pretraining or representation alignment. Such integration may be useful for heterogeneous conditions including HF, cardiomyopathies, and VHD, for which ECG manifestations overlap and definitive assessment often requires additional clinical information (23–25). However, multimodal studies should compare ECG-only, non-ECG-only, and integrated models to determine whether ECG representations provide incremental information rather than reproduce existing clinical context.

Overall, architecture selection should be guided by the electrophysiological substrate, available ECG format, intended clinical use, and downstream action rather than architectural novelty. Raw-signal models preserve waveform amplitude and temporal information, whereas image-based models provide a practical option when ECGs are available only as scans or PDF files but may learn grids, labels, device markers, or institution-specific formatting. No representation strategy is universally optimal. The following disease-specific sections therefore evaluate not only what each model can detect, but also whether it addresses a defined clinical use, relies on a credible reference standard, generalizes across populations, institutions, and devices, produces interpretable and actionable outputs, and improves clinical workflow or patient outcomes (Figure 3).

**Figure 3:**
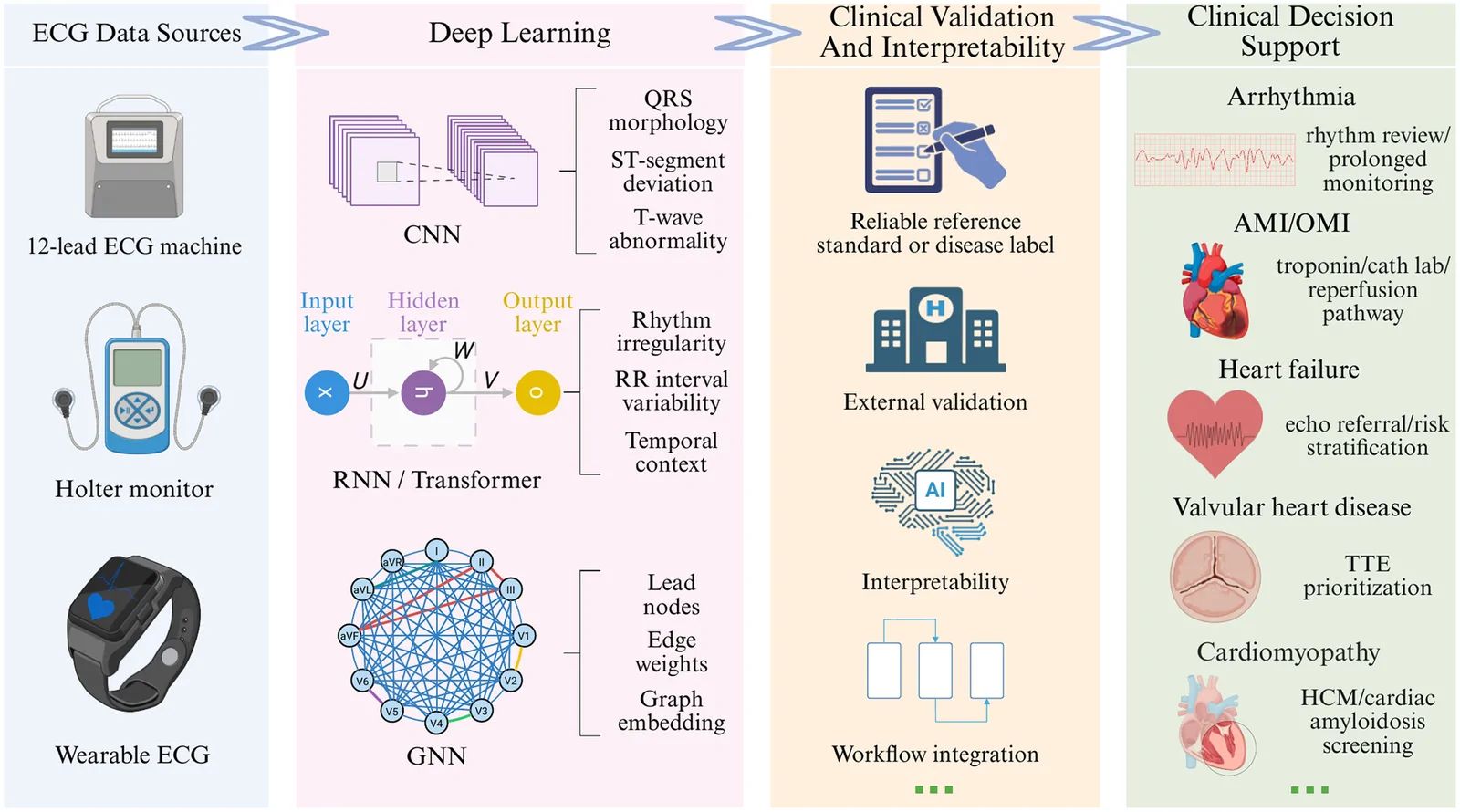
Translational pathway from AI-ECG representation learning to clinical decision support. AI-ECG systems extract morphological, temporal, and cross-lead representations from 12-lead ECGs, ambulatory recordings, and wearable signals. Their translation into arrhythmia review, AMI/OMI triage, HF or left ventricular dysfunction screening, VHD assessment, and cardiomyopathy detection requires reliable disease labels, external validation, clinically meaningful operating thresholds, interpretable outputs, explicit downstream actions, and prospective evaluation of workflow and patient outcomes. Created in BioRender. Chen, H. (2026) https://BioRender.com/9t1gp0p

## 5 Clinical applications of AI-ECG in cardiovascular diseases

### 5.1 Arrhythmia detection and prediction

AI-ECG in arrhythmia care addresses three distinct tasks: classifying rhythms present on the recording, identifying patients with sinus-rhythm ECGs who may benefit from prolonged AF monitoring, and recognizing coexisting rhythm or conduction abnormalities. Because these tasks differ in label reliability and downstream action, their reported performance cannot be interpreted using the same evidentiary standard.

AF detection and prediction illustrate this distinction. AF is characterized by absent *P* waves, fibrillatory activity, and irregular RR intervals (26), but PAF may not be captured on a standard 10-second ECG (27). Attia et al. (27) showed that a ResNet-based CNN could identify patients at risk of PAF from sinus-rhythm ECGs recorded before clinical diagnosis, suggesting that DL may capture ECG signatures consistent with latent atrial remodeling or AF susceptibility. Yuan et al. (28) extended this concept in more than 900,000 outpatient sinus-rhythm ECGs from the U.S. Veterans Affairs system and reported external testing across additional Veterans Affairs sites and a non–Veterans Affairs academic center. This study is stronger than many benchmark-based classification studies because it evaluates a clinically plausible use case: selecting patients for more intensive AF monitoring. Nevertheless, even this evidence should be viewed as risk enrichment rather than definitive diagnosis. The reference standard depends on subsequent AF detection in clinical records, so undiagnosed AF in controls and differences in monitoring intensity may affect label validity. Moreover, prospective evidence is still needed to determine whether DL-guided screening reduces stroke, thromboembolism, or unnecessary monitoring.

For multi-class arrhythmia diagnosis, recent architectures increasingly attempt to encode the spatial structure of 12-lead ECGs rather than treating leads as independent channels. Andayeshgar et al. (29) explicitly modeled inter-lead relationships using mutual information as the adjacency matrix of a graph convolutional network (GCN), whereas Yaser et al. (30) used a temporal convolutional network–GCN framework to construct cycle-level graph representations from ECG signals. Together, these studies suggest that graph-based representations can improve arrhythmia classification, although the physiological meaning of the graph differs across models. However, their evidence remains closer to technical feasibility than clinical translation. Most evaluations rely on public datasets and cross-validation, and the reference labels inherit the annotation practices and disease definitions of those datasets. High accuracy on a curated rhythm database may not generalize to emergency departments, ambulatory devices, wearable single-lead recordings, noisy signals, paced rhythms, or patients with overlapping cardiovascular disease. Thus, graph-based and spatially informed DL models are methodologically promising, but their clinical reliability requires external validation across institutions, devices, acquisition protocols, and patient subgroups.

Multi-label arrhythmia classification addresses a more clinically realistic problem: patients may have concurrent rhythm, conduction, and morphological abnormalities. Jiang et al. (31) modeled label dependencies with a gated recurrent unit feature extractor and GCN, while Li et al. (32) used a binary-relevance strategy with a Bi-LSTM backbone. Liu et al. (33) further highlighted two clinically important issues often missed by accuracy-focused models: class imbalance and unequal misclassification cost. For example, confusing AF with atrial flutter is not equivalent to classifying AF as normal sinus rhythm, because the downstream clinical risk and management implications differ. Yang et al. (34) also showed that multi-view, multi-scale modeling and knowledge distillation can improve multi-label ECG classification across benchmark tasks. Automated classification of arrhythmias present on the ECG is therefore technically mature in benchmark settings, whereas prediction of occult or future AF remains a strategy for selecting patients for prolonged monitoring rather than establishing a diagnosis. Graph-based and multi-label systems remain primarily benchmark-validated methods, and their clinical value depends on device-diverse external testing and evidence that they reduce missed clinically important arrhythmias without increasing review burden (Table 1).

### 5.2 Acute myocardial infarction and occlusion-focused triage

ECG remains a first-line tool for evaluating suspected AMI because myocardial ischemia may produce ST-segment and T-wave abnormalities that inform acute diagnostic and reperfusion pathways (35). Within current reperfusion pathways for ST-segment elevation myocardial infarction (STEMI), ST-segment elevation in the appropriate clinical context remains the principal electrocardiographic trigger for urgent catheterization; however, this morphology-based criterion captures only part of the pathophysiological spectrum of acute coronary occlusion. AI-ECG analysis has therefore been increasingly investigated not only as an automated interpretation tool, but also as a clinically actionable decision-support layer for emergency and prehospital triage, where early recognition directly affects time to reperfusion (36). In a pragmatic randomized trial, AI-ECG-assisted STEMI triage reduced median door-to-balloon time in emergency department patients and shortened ECG-to-balloon time across emergency and inpatient settings, supporting its potential to mitigate workflow-related delays in primary percutaneous coronary intervention pathways (37). Beyond rule-in activation for reperfusion pathways, AI-ECG has also been explored for early rule-out and low-risk triage in suspected AMI. In the prospective multicenter ROMIAE study, a ResNet-based model using only the initial 12-lead ECG achieved an AUROC of 0.878 and, at the predefined low-risk threshold, reached 99.6% sensitivity and 99.1% negative predictive value. This supports AI-ECG as a rapid front-line triage layer in emergency AMI pathways, while still requiring integration with biomarkers and clinical judgment (38).

Earlier AI-ECG studies in this field primarily focused on automated MI detection and anatomical localization, whereas recent work has increasingly shifted toward emergency triage and occlusion-oriented decision support. Xiong et al. (39) developed a DenseNet-based multi-lead framework that exploited both intra-lead morphology and inter-lead correlations, demonstrating that 12-lead ECG signals contain spatially distributed information relevant to infarct localization. Subsequent models moved beyond binary MI detection toward clinically meaningful territory classification. For example, Chen et al. (18) proposed a multi-domain feature fusion CNN that integrated raw time-domain signals, two-dimensional spectral representations, and time–frequency features, achieving fine-grained localization across 11 MI categories with 84.86% localization accuracy under inter-patient validation. Similarly, Kim et al. (40) introduced a guideline-informed two-stage framework in which Faster R-CNN first detected ST-segment abnormalities, followed by weighted ensemble learning to assign infarction to clinically used territories, including anterior, lateral, inferior, and left main involvement.

These localization studies remain primarily supported by database-derived diagnostic labels and retrospective validation. Their main translational value lies in linking model outputs to lesion-specific lead patterns and clinically recognizable infarct territories, thereby providing a more interpretable bridge between AI prediction and cardiologist-style ECG reasoning. However, their clinical utility remains less established than that of AI-ECG applications evaluated directly within acute triage or reperfusion pathways.

Nevertheless, the conventional STEMI/NSTEMI paradigm has important limitations when the clinical target is acute coronary occlusion rather than ST elevation alone. A recent diagnostic accuracy meta-analysis reported that ST-segment elevation had a pooled sensitivity of only 43.6% for detecting acute coronary occlusion, despite high specificity (41). This diagnostic gap has motivated the OMI framework, which reframes urgent reperfusion decisions around angiographically confirmed occlusion or flow-limiting culprit lesions rather than isolated ST-segment criteria (41, 42). Within this context, DL models have shown promise in identifying subtle or atypical ECG signatures of OMI using clinically stronger reference standards. Riek et al. (43) developed ECG-SMART-NET, a clinically informed architecture that first learns lead-specific temporal features and then cross-lead spatial patterns, achieving a test AUC of 0.953 for OMI detection in a multisite real-world dataset. In an international evaluation, an AI-ECG model detected angiographically defined OMI with an AUC of 0.938 and markedly higher sensitivity than standard STEMI criteria, while maintaining high specificity and performance comparable to ECG experts (42).

From a translational perspective, AI-ECG has reached workflow-level evidence for STEMI activation and prospective multicenter evidence for AMI rule-out, whereas OMI models remain at the external diagnostic-validation stage. The next step is therefore not another retrospective discrimination study, but prospective interventional evaluation of whether OMI-guided activation improves reperfusion decisions without excessive false catheterization-laboratory activation (Table 2).

### 5.3 Heart failure screening and risk stratification

HF and related left ventricular dysfunction remain major contributors to mortality, recurrent hospitalization, and healthcare burden in patients with cardiovascular disease (44). AI-ECG studies in this field address several distinct tasks: staging established HF, screening for reduced left ventricular ejection fraction (LVEF) or heart failure with reduced ejection fraction (HFrEF), identifying heart failure with preserved ejection fraction (HFpEF), predicting future HF, and directing confirmatory testing within clinical workflows. These tasks should not be treated as a single diagnostic problem because they use different reference standards and trigger different downstream actions.

HF stage classification generally relies on composite clinical definitions, whereas reduced-LVEF screening uses echocardiography as a more objective and actionable reference standard. Li et al. (45) combined lead II ECG segments with clinical variables to classify five HF stages and reported high retrospective performance; however, the model was not externally validated and its intended downstream action was not clearly defined. Attia et al. (46) externally validated a CNN for left ventricular systolic dysfunction defined as LVEF ≤35%, achieving an AUC of 0.82. At the reported operating threshold, however, sensitivity was 26.9% and specificity was 97.4%, indicating a high-specificity risk-enrichment strategy rather than a broadly sensitive screening test. Hou et al. (47) extended reduced-LVEF detection to ECG images using LVEF ≤50% as the endpoint and reported an AUC of 0.965.

Clinically, reduced-LVEF models are best interpreted as screening or risk-enrichment tools that prioritize confirmatory echocardiography rather than replace it. Their usefulness depends not only on discrimination but also on the intended operating threshold, disease prevalence, and local capacity for downstream imaging. The low sensitivity reported at some high-specificity thresholds further illustrates why model performance must be interpreted in relation to the intended screening pathway.

HFrEF detection has a relatively direct clinical pathway because a positive result can prompt echocardiography and treatment assessment. Choi et al. (48) evaluated DeepECG-HFrEF in patients with acute symptomatic HF and reported an AUC of 0.844. Model-positive patients also had less favorable ECG and echocardiographic characteristics and worse 5-year survival. Because the cohort already consisted of symptomatic patients, these findings support HFrEF phenotyping and prognostic enrichment more directly than population-level screening. In a prospective multicenter study, Bachtiger et al. (49) used an ECG-enabled stethoscope to acquire single-lead recordings at several anatomical positions and achieved an AUROC of 0.91 for LVEF ≤40%, supporting point-of-care prioritization of echocardiography.

HFpEF is a less stable prediction target because preserved LVEF does not exclude disease and no single definitive reference standard is available. Diagnosis instead integrates symptoms, echocardiographic measures, natriuretic peptides, and structured scores such as HFA-PEFF. Hong et al. (50) trained a CNN to identify patients with HFA-PEFF scores ≥5 and achieved an AUROC of 0.81. Positive predictions were associated with higher 5-year risks of cardiac death and HF hospitalization. Nevertheless, the single-center design and reliance on a composite score mean that the model currently provides HFpEF risk enrichment rather than diagnostic confirmation.

Longitudinal and multimodal approaches remain less clinically mature. Chen et al. (23) used ECG-report alignment and dual-attention modeling to predict incident HF in UK Biobank hypertension and myocardial infarction cohorts, but the reported C-index values were modest and the model has not been evaluated in a prospective decision pathway. In a multimodal model, Park et al. (24) increased the AUC for HFrEF detection from 0.896 with ECG alone to 0.950 by adding age, N-terminal pro-B-type natriuretic peptide, and serum sodium. This improvement supports context-aware decision support, although part of the gain derives from established non-ECG predictors and should not be attributed to ECG representation alone.

Workflow-level evidence is concentrated in reduced-LVEF screening. In a pragmatic randomized controlled trial involving non-cardiology inpatients, Tsai et al. (51) implemented an AI-ECG alert for reduced LVEF. The intervention increased newly diagnosed reduced LVEF among AI-identified high-risk patients without increasing overall echocardiography use, raised the positive predictive value of echocardiography from 20.2% to 34.2%, and increased cardiology consultation from 23.5% to 29.3% (Table 3).

This randomized workflow evidence places reduced-LVEF screening among the most mature HF applications because it combines an objective imaging reference standard, an explicit downstream action, and demonstrated improvement in diagnostic yield. By contrast, HFrEF point-of-care detection remains promising, whereas HFpEF identification and long-term HF prediction remain primarily risk-enrichment tasks. No included interventional study has demonstrated improved HF treatment initiation, hospitalization, or survival.

### 5.4 Valvular heart disease screening

Given the resource requirements of population-wide echocardiographic screening, AI-ECG is best used to prioritize confirmatory echocardiography in patients at risk of valvular heart disease rather than as a replacement for echocardiography (52). Most available studies used TTE-defined moderate-to-severe VHD as the reference standard, providing a clinically acceptable disease label; however, label reliability may still be influenced by the ECG–TTE time interval, echocardiographic grading variability, and the retrospective nature of data extraction. Among VHD phenotypes, aortic stenosis (AS) and mitral regurgitation (MR) have been most extensively studied. In AS, pressure overload–induced ventricular hypertrophy and repolarization abnormalities provide a plausible electrophysiological basis for AI-ECG detection (52). For MR, Kwon et al. (53) developed a CNN-based model using echocardiography-confirmed moderate-to-severe MR as the endpoint and reported AUROCs of 0.816 and 0.877 in internal and external validation, respectively, with saliency maps highlighting *P*- and T-wave regions in MR cases and QRS complexes in non-MR cases.

External validation across institutions and populations has strengthened the evidence base, although device-level generalizability and performance across acquisition settings remain insufficiently established. Lin et al. (54) developed AI-ECG models for AS, aortic regurgitation (AR), MR, tricuspid regurgitation (TR), and pulmonary regurgitation using ECG–TTE pairs and validated them in a community hospital cohort, achieving AUCs above 0.77–0.84 and NPVs exceeding 94.3%, supporting AI-ECG as a rule-out and referral-support tool for multiple VHDs. Elias et al. (52) similarly demonstrated that DL-enabled ECG could detect moderate-to-severe AS, AR, and MR in a multicenter cohort, although screening performance was highly dependent on disease prevalence and positive predictive value remained limited.

Beyond cross-sectional detection, AI-ECG may also provide prognostic information. Lin et al. (54) reported that 37.5%–51.7% of false-positive predictions had significant echocardiographic abnormalities. The presence of other echocardiographic abnormalities among model-positive patients suggests that the algorithm may capture broader structural remodeling; however, these cases remain false positives for the prespecified VHD endpoint. Liang et al. (55) further extended this concept by using survival neural networks to predict future significant MR, AR, and TR, with transnational external validation and marked risk separation across predicted risk groups. VHD therefore remains a validation-stage screening application: AI-ECG can prioritize TTE and enrich cohorts at risk of future valve disease, but prevalence-dependent positive predictive value and the absence of prospective workflow evidence preclude population-wide or stand-alone use. As a multimodal example, Nagai et al. (56) further showed that combining ECG with chest radiography improved aortic stenosis detection compared with either modality alone, although the incremental gain was modest and the evaluation remained retrospective (Table 4).

### 5.5 Hypertrophic cardiomyopathy screening

HCM is an appropriate target for AI-ECG opportunistic screening because it is frequently underdiagnosed, requires imaging-based confirmation, and has important downstream implications for family screening, sudden cardiac death risk stratification, and disease-specific management. Ko et al. (57) first demonstrated that a CNN trained on standard 12-lead ECGs could detect HCM with high diagnostic performance, achieving an AUC of 0.96, sensitivity of 87%, and specificity of 90% in the testing dataset. However, this model was developed in a single referral-center cohort, and the authors emphasized the need for further external validation. The reference standard is relatively strong when HCM labels are based on expert review, echocardiography, or cardiac magnetic resonance (CMR), but label reliability may still vary across studies. For example, Siontis et al. (58) defined HCM using ESC and ACC/AHA criteria, including left ventricular wall thickness thresholds on echocardiography or CMR in the absence of alternative causes of hypertrophy, whereas Sangha et al. (10) used imaging-based HCM features in development and CMR-based labels in external validation cohorts, but relied on diagnosis codes in the UK Biobank cohort. These differences are clinically important because HCM can overlap phenotypically with hypertensive left ventricular hypertrophy (LVH), aortic stenosis, athletic remodeling, and cardiac amyloidosis.

International and cross-format testing has moved AI-ECG for HCM beyond single-center proof of concept, although transportability remains dependent on population, device, and label characteristics. Siontis et al. (58) externally validated the Mayo Clinic AI-ECG algorithm in international cohorts from Switzerland, the United Kingdom, and South Korea, reporting an overall AUC of 0.922, diagnostic accuracy of 86.9%, sensitivity of 82.8%, and specificity of 87.7%. Sangha et al. (10) further showed that an ECG image–based DL model could maintain robust performance across image layouts and external datasets, with AUROCs of 0.95 in internal testing, 0.94 in MIMIC-IV, 0.92 in AUMC, and 0.91 in UK Biobank. Goto et al. (59) highlighted why cross-institution validation is essential: single-institution ECG models showed excellent internal discrimination but degraded when transferred across countries, whereas federated learning improved generalizability for both ECG and echocardiography models. These findings suggest that AI-ECG may support HCM screening across heterogeneous data environments, but performance still depends on population composition, disease prevalence, ECG acquisition format, device source, and the quality of downstream confirmatory testing.

The clinical actionability of HCM prediction depends on using AI-ECG as a triage signal rather than a stand-alone diagnosis. Sangha et al. (10) used gradient-weighted class activation mapping to localize discriminative regions mainly to anterior and lateral leads, especially V4 and V5, providing partial interpretability and supporting physiological plausibility. Nevertheless, heatmap-based interpretation cannot fully explain which electrophysiological features drive the prediction. Clinically, a positive AI-ECG result should therefore prompt confirmatory echocardiography or CMR, review of family history, consideration of genetic counseling, and subsequent risk stratification, rather than direct disease labeling. Love et al. (60) provided the most direct workflow evidence in a multicenter prospective implementation study: among 145,848 screened ECGs, 4,348 alerts were generated, 69% were viewed by users, and 17 newly diagnosed HCM cases were identified among 217 enrolled patients. The median time from ECG to diagnostic imaging indicating HCM was 7.5 days. These data support feasibility and diagnostic workflow acceleration, but evidence that AI-ECG improves long-term outcomes, cascade screening uptake, treatment allocation, or sudden cardiac death prevention remains limited. HCM is therefore one of the more mature structural-disease applications: international validation and prospective implementation support opportunistic screening, but no included study has demonstrated improved cascade screening, treatment allocation, sudden cardiac death prevention, or long-term outcomes (Table 5).

### 5.6 Cardiac amyloidosis screening

Within the spectrum of hypertrophic phenocopies, cardiac amyloidosis (CA) is an appropriate target for AI-ECG because conventional ECG findings are often insensitive and its phenotype overlaps with HCM, hypertensive heart disease (HHD), and HFpEF. Accordingly, the intended clinical use is referral support in patients with unexplained LVH, HF, or extracardiac transthyretin amyloidosis (ATTR) red flags, rather than autonomous disease confirmation. Schrutka et al. (61) linked electrocardiographic imaging-derived low voltage and altered activation to a visually interpretable 12-lead ECG algorithm; training cardiologists on this rule increased the AUC from 0.69 to 0.97, supporting an interpretable decision-support approach. Haimovich et al. (62) extended the task from binary detection to LVH etiology classification, showing that the 12-lead LVH-Net achieved an AUROC of 0.95 for CA and that lead I and lead II models retained favorable discrimination.

Despite favorable discrimination, the evidence remains limited by cohort composition and transportability. The Schrutka study used a small, single-center reader-training design, while the Haimovich validation hospitals belonged to one referral health system and the cohort was predominantly older and White. Although CA and HCM labels were adjudicated using imaging and pathology data, generalizability to wearable ECGs, other healthcare systems, and specific amyloid subtypes remains uncertain.

Reference standards and validation designs remain heterogeneous. Harmon et al. (63) defined immunoglobulin light-chain (AL) amyloidosis using biopsy and ATTR using biopsy or positive technetium-99 m pyrophosphate scintigraphy with additional criteria for cardiac involvement. In 440 post development CA cases and 6,600 matched controls, the model achieved an AUC of 0.84, but performance was lower in Hispanic patients (AUC 0.66) and ECGs with LVH (AUC 0.75) or left bundle branch block (AUC 0.76). This was nevertheless an internal temporal validation at a single institution, and occult CA in controls could not be excluded. González-López et al. (64) used European Society of Cardiology-consistent transthyretin amyloid cardiomyopathy (ATTR-CM) diagnoses and controls with negative technetium-99m–labeled 3,3-diphosphono-1,2-propanodicarboxylic acid scintigraphy plus clinical assessment. Willem AI achieved an AUC of 0.88, sensitivity of 80.7%, and specificity of 78.5%, but sensitivity decreased to 68.4% in asymptomatic patients; the study remained single-center, predominantly Caucasian, and without completed external validation. Zhang et al. (25) provided the broadest multicenter evidence for multimodal triage in this section. Their seven-variable model combined ECG, echocardiographic, and clinical information and achieved an externally validated AUC of 0.96 for CA across 10 Chinese hospitals. However, this result demonstrates the transportability of an integrated diagnostic model rather than an ECG-only algorithm.

The models also differ in whether they provide a clinically usable explanation or only a probabilistic risk score. Schrutka et al. (61) provided a mechanistic link between surface ECG patterns and electroanatomical abnormalities, Zhang et al. (25) used SHapley Additive exPlanations and a simplified score, and Haimovich et al. (62) associated higher predicted CA risk with lower voltage and wider QRS complexes; by contrast, González-López et al. (64) identified the lack of model explainability as a current limitation of Willem AI.

A positive prediction should therefore trigger structured clinical review and confirmatory echocardiography, CMR, monoclonal protein testing, bone scintigraphy, or biopsy according to the suspected subtype, rather than direct CA labeling. Zhang et al. (25) embedded the model in a WeChat program and proposed a three-tier triage pathway, but the simplified score had 75.4% sensitivity in external validation and the workflow was not prospectively tested for diagnostic yield or time savings. González-López et al. (64) similarly proposed adjustable thresholds and confirmatory testing, whereas Harmon et al. (63) called for prospective clinical integration studies. Cardiac amyloidosis therefore remains a suspicion-raising application for selected patients with unexplained LVH or HF. Existing models can support subtype-directed confirmatory testing, but phenotype-restricted cohorts, incomplete device and ethnic transportability, and the absence of prospective workflow evaluation limit broader screening (Table 6).

## 6 Discussion

### 6.1 Translational maturity across applications

This review shows that translational maturity varies substantially across AI-ECG applications and is determined less by peak discrimination than by the alignment of three practical elements: a credible reference standard, an explicit downstream clinical action, and a measurable workflow or clinical endpoint. Using the review-specific evidence stages defined in the Methods, the applications that have progressed furthest are generally those in which model output can be linked directly to an established care pathway.

Among the applications reviewed, AI-assisted STEMI triage and AMI rule-out are among the most advanced, supported by randomized workflow evidence and prospective multicenter validation, respectively. Randomized evidence suggests that AI-assisted STEMI identification can reduce treatment delays, while prospective multicenter evidence supports rapid low-risk triage for suspected AMI (37, 38). OMI-oriented models address an important limitation of STEMI criteria and use angiographically stronger reference standards. However, most OMI evidence remains diagnostic rather than interventional. It has not established whether AI-guided activation improves reperfusion decisions or patient outcomes without causing excessive false activations.

Screening for reduced left ventricular ejection fraction represents another comparatively advanced application. These models have a clear downstream action, namely confirmatory echocardiography, and some have undergone external or prospective evaluation (46, 49, 51). A pragmatic randomized study demonstrated improved diagnostic yield and cardiology consultation without increasing overall echocardiography use (51). This constitutes stronger evidence of clinical utility than retrospective AUROC alone. Nevertheless, effects on treatment initiation, hospitalization, mortality, and cost-effectiveness remain uncertain.

HCM screening occupies an intermediate but advancing position. Imaging-based labels, international external validation, and multicenter prospective implementation strengthen its evidence base (10, 58, 60). These studies support AI-ECG as an opportunistic screening and referral tool. They do not yet show that screening improves cascade testing, treatment allocation, sudden cardiac death prevention, or long-term outcomes.

The evidence for AF is task-dependent. Automated detection of AF present on an ECG is technically mature in benchmark settings, although performance may not transfer to noisy clinical or wearable recordings. Predicting future or occult AF from sinus rhythm has greater translational uncertainty. Large-scale external testing supports its use for risk enrichment (27, 28), but the prediction is not diagnostic. Its value depends on whether AI-guided monitoring improves selection for prolonged rhythm assessment and ultimately prevents stroke without producing unnecessary surveillance.

VHD screening, HFpEF identification, long-term HF risk prediction, and cardiac amyloidosis screening remain less mature. Their lower maturity reflects limited prospective implementation and outcome evidence rather than uniformly weak discrimination (23, 25, 50, 52, 55, 63). VHD models may help prioritize echocardiography, while cardiac amyloidosis models may raise suspicion in patients with unexplained hypertrophy or heart failure (64). None of these applications has shown consistent benefit in prospective, device-diverse workflows. Patient-outcome evidence remains uncommon across all reviewed disease domains (Table 7).

**Table 7:** Cross-application comparison of translational maturity in AI-ECG.

Clinical application	Intended clinical use	Reference standard	Highest evidence stage	Linked clinical action	Key strength	Key limitation
STEMI reperfusion triage	Emergency rule-in and reperfusion-pathway activation	Established STEMI diagnosis within an acute reperfusion pathway	Controlled workflow evaluation (pragmatic randomized trial)	Expert review and urgent catheterization-laboratory or reperfusion activation	Randomized evidence of improved workflow timeliness; direct link to reperfusion pathway	Patient-level outcome benefit not established; false activation remains relevant
AMI rule-out	Early low-risk triage in suspected AMI	Serial biomarkers, clinical adjudication, and follow-up	Prospective multicenter diagnostic validation	Continued biomarker-based assessment or expedited low-risk pathway	Prospective multicenter validation; very high sensitivity/NPV at prespecified threshold	Effect on discharge strategy, resource use, and outcomes not tested
OMI detection	Occlusion-oriented reperfusion triage	Angiographic occlusion or culprit lesion	Independent transportability (international external validation)	Urgent coronary assessment or reperfusion review	Angiographic reference standard; international external validation	No prospective interventional evidence
Reduced-LVEF screening	Opportunistic screening and referral prioritization	Echocardiographic LVEF	Controlled workflow evaluation	Echocardiography and cardiology review	Objective echocardiographic endpoint; randomized workflow evidence	Effects on HF hospitalization and mortality remain unestablished
HCM screening	Opportunistic screening and referral support	Echocardiography, CMR, and guideline-based adjudication	Prospective implementation	Imaging, family-history review, and genetic counseling	International validation + prospective implementation	Incomplete downstream evaluation of all alerts; long-term benefit unestablished
Future or occult AF prediction	Risk enrichment for prolonged monitoring	Subsequent clinically documented AF	Independent transportability (external validation)	Extended rhythm monitoring	Large-scale external validation	Prediction depends on subsequent ascertainment; benefit of AI-guided monitoring unknown
VHD screening	Echocardiography prioritization and risk stratification	TTE-defined disease severity	Independent transportability (external validation)	Confirmatory echocardiography	Echocardiographic reference + external validation	PPV is prevalence-dependent; no prospective workflow evidence
HFpEF and long-term HF prediction	Diagnostic or prognostic risk enrichment	Composite scores or follow-up outcomes	Patient-level model development/cohort validation	Imaging, biomarker assessment, or closer follow-up	Clinically important unmet need	Composite/heterogeneous reference standard; mainly retrospective
Cardiac amyloidosis screening	Suspicion raising and referral support	Biopsy, scintigraphy, imaging, and subtype-specific criteria	Independent transportability for a multimodal model; ECG-only evidence remains mainly single-center	Imaging, monoclonal protein testing, scintigraphy, or biopsy	Pathology/imaging-based labels in selected studies	Small/referral cohorts, ethnic/device transportability concerns, no ECG-only prospective workflow evidence

The evidence stage represents the highest stage reached by at least one study within each application and does not imply that all models have achieved the same level of validation. Translational maturity is distinct from risk of bias and represents a structured narrative assessment rather than a formal quantitative grading system.

### 6.2 Intended use and reference-standard reliability

Intended use should determine model development and evaluation because screening, diagnostic triage, risk stratification, monitoring, and workflow prioritization involve different populations, error costs, thresholds, and downstream actions. A model developed to select patients for echocardiography should not be presented as an autonomous diagnostic system. Similarly, an AF risk score derived from sinus-rhythm ECGs should indicate a need for additional monitoring rather than established AF (26–28).

Expert-adjudicated rhythm labels can provide a strong standard for arrhythmias visible on the recording. However, labels for future AF depend on monitoring intensity and subsequent clinical detection. Undiagnosed AF among controls may introduce outcome misclassification. A model may therefore predict both atrial susceptibility and the likelihood of future clinical ascertainment rather than the biological presence of arrhythmia alone (27, 28). AMI labels based on serial biomarkers and clinical adjudication are stronger than isolated diagnostic codes (38). For OMI, angiographic occlusion or a flow-limiting culprit lesion is more closely aligned with the intended reperfusion decision than ST elevation alone (43). Structural and functional diseases introduce other uncertainties. Echocardiographic LVEF provides a relatively objective reference for reduced systolic function, although thresholds and ECG-to-imaging intervals vary (46–48). VHD labels depend on echocardiographic grading, reader variability, and temporal alignment between ECG and imaging (52, 55, 56). HCM labels are strongest when guideline criteria, echocardiography, CMR, and expert adjudication exclude phenocopies. Diagnostic codes alone are less reliable (10, 57–60). HFpEF is particularly difficult because composite scores combine imaging, biomarkers, symptoms, and clinical context rather than a single definitive test (44, 50). Cardiac amyloidosis illustrates the consequences of heterogeneous labeling. Biopsy, bone scintigraphy with appropriate exclusion of monoclonal disease, and subtype-specific clinical criteria provide stronger evidence than administrative diagnoses. Label quality also affects control groups because occult disease may remain undetected (61–63). Transparent reporting of label definitions, adjudication procedures, test timing, and missing data is therefore necessary for interpreting model performance.

For structural disease, assigning a patient-level diagnosis to every beat or segment creates weak labels, whereas distributing segments from the same patient across training and test sets causes data leakage. Patient-level separation should therefore be a minimum requirement for credible AI-ECG evaluation.

### 6.3 External validation, prospective evaluation, and generalizability

External validation and prospective validation address different questions and should not be used interchangeably. External validation evaluates transportability by testing a fixed model in data that are independent of the development environment, such as another institution, country, device, or population; it may still be performed retrospectively. Prospective validation instead evaluates a prespecified model on patients or ECGs collected after the evaluation protocol has been defined, often within an intended clinical workflow, but it may occur within the same institution in which the model was developed. Thus, a study can be external but retrospective, prospective but not external, or both prospective and external. For example, international testing of OMI models primarily provides external evidence of diagnostic transportability, whereas the multicenter prospective HCM implementation study evaluates how a fixed alert functions in real clinical practice. Pragmatic randomized evaluations of STEMI triage and reduced-LVEF screening go a step further by testing whether AI-assisted workflows change clinical processes relative to usual care.

Generalizability cannot be established by random internal splitting alone. A useful validation hierarchy begins with patient-level internal testing, followed by temporal validation, independent institutional validation, multicenter evaluation, and testing across countries, populations, devices, and acquisition formats. Performance should also be examined in clinically relevant subgroups rather than only in the pooled cohort. Several HCM studies illustrate the value of this progression. International validation has shown that performance can remain favorable across different populations (10, 58). Federated learning studies have also demonstrated that models with excellent local performance may degrade after transfer between institutions (59). These findings caution against treating internal discrimination as evidence of transportability.

The same concern applies to public arrhythmia benchmarks. Curated recordings may underrepresent noise, paced rhythms, comorbidity, overlapping abnormalities, and wearable-device artifacts. Models trained on one database may also learn annotation conventions rather than disease physiology. External validation is clinically informative only when the test cohort approximates the intended deployment population, acquisition system, disease prevalence, and workflow. Testing another sample from the same data-generating environment provides weaker evidence of transportability. Engineering heterogeneity is clinically consequential. Sampling frequency, filtering, lead configuration, signal duration, device firmware, proprietary formats, and image layouts can alter model inputs without changing the patient's disease. A model may therefore fail because it learned device-specific or institution-specific features. Standardized ECG data formats can reduce avoidable variation, although implementation differences remain substantial (65).

External validation should reproduce the complete preprocessing pipeline and test robustness to clinically relevant differences in sampling frequency, filtering, lead configuration, signal duration, and signal format (17, 18, 65). A high AUROC without independent testing across relevant populations and acquisition systems cannot support clinical use by itself.

### 6.4 From discrimination to clinical actionability

AUROC summarizes discrimination across possible thresholds but does not describe calibration, predictive value, diagnostic yield, downstream workload, or the consequences of errors at the selected operating threshold. This distinction is especially important in low-prevalence screening. Even a model with high sensitivity and specificity may generate many false-positive alerts when the target disease is uncommon. Positive predictive value determines how many flagged patients are likely to have disease. Negative predictive value informs the safety of rule-out strategies. Both depend on prevalence and must be reported in the intended population.

Operating thresholds should reflect task-specific error costs rather than a universal performance target. An AMI rule-out pathway may prioritize sensitivity and negative predictive value because missed disease carries substantial risk. HCM or cardiac amyloidosis screening must balance sensitivity against imaging, referral, anxiety, and incidental findings. OMI detection must balance missed occlusions against unnecessary catheterization laboratory activation. These examples illustrate why operating thresholds must balance missed disease against unnecessary testing or intervention.

Clinical actionability is more informative than discrimination alone when assessing translational value. A positive reduced-LVEF screen can trigger targeted echocardiography and cardiology review (49, 51). Suspected HCM may prompt echocardiography or CMR, family-history review, and genetic counseling (60). A cardiac amyloidosis alert may initiate subtype-appropriate confirmatory testing (25, 63). Suspected AMI or OMI may accelerate biomarker testing, coronary assessment, or reperfusion review (37, 38, 42, 43). The output should therefore be linked to an explicit action pathway before deployment. Predictions without an agreed clinical response risk creating alert fatigue or duplicating existing tests.

Multimodal models require additional caution. Improved performance after adding clinical variables may reflect comorbidities, testing patterns, or prior diagnostic decisions rather than disease-specific ECG information. Multimodal studies should compare ECG-only, non-ECG-only, and integrated models to quantify the incremental contribution of the ECG (24, 25, 56). Otherwise, such systems are better described as context-aware decision support than as autonomous ECG interpretation.

### 6.5 Workflow integration and patient outcomes

Only a small subset of AI-ECG applications has progressed beyond external validation: randomized evaluations have demonstrated workflow benefits for STEMI triage and reduced-LVEF screening, whereas prospective HCM implementation has established feasibility without demonstrating improved long-term outcomes (37, 51, 60). These studies provide evidence of implementation feasibility, but they do not establish broad outcome benefits across AI-ECG applications.

Workflow outcomes include time to diagnosis or treatment, clinician workload, alert acceptance, and test utilization. Decision-related outcomes include referral, confirmatory testing, treatment initiation, and changes in diagnostic confidence. Patient-level evaluation should examine hospitalization, mortality, stroke prevention, quality of life, cost-effectiveness, and unintended harms. Disease-specific outcomes should also be considered. Relevant examples include reperfusion delay, HF hospitalization, timely initiation of disease-modifying therapy, and prevention of AF-related thromboembolism. Such outcomes should be prespecified and linked to the model's intended use.

Implementation can remain incomplete even when diagnostic performance is favorable, because alert uptake and downstream follow-up may be limited (51, 60). Excessive false positives may increase workload, while poorly designed rule-out pathways may create false reassurance. Workflow studies should specify where the model operates, who receives its output, what action is expected, and how disagreements between the model and clinicians are resolved.

### 6.6 Physiological fidelity, interpretability, and clinician oversight

Physiological fidelity connects engineering reliability with clinical safety. Denoising may alter diagnostically relevant waveform components when noise suppression is optimized without explicit assessment of physiological fidelity (66). Fixed beat windows may provide incomplete waveform coverage when cardiac-cycle duration varies substantially, motivating morphology-aware segmentation strategies (67). Resampling and filtering can also alter waveform morphology. Preprocessing should therefore be evaluated according to preservation of clinically relevant signals, not only reconstruction error. Clinical deployment must also account for recordings that are incomplete or not of diagnostic quality. Motion artifact, baseline wander, poor electrode contact, lead reversal or misplacement, signal clipping, and missing leads can alter waveform morphology or prevent a model from generating a valid output (65, 66). Models developed predominantly on curated, high-quality recordings may therefore show optimistic performance when transferred to emergency, ambulatory, or wearable settings. Importantly, excluding non-analyzable ECGs from performance calculations may inflate reported accuracy and obscure the proportion of patients for whom the system provides no usable result. External and prospective evaluations should consequently report ECG rejection rates, reasons for model failure, performance under missing-lead or degraded-signal conditions, and, where relevant, intention-to-diagnose performance.

Interpretability should similarly extend beyond saliency maps. Heatmaps localize influential regions but do not, by themselves, establish physiological causality or exclude reliance on confounding features. An explanation is clinically useful only if it changes confidence, identifies a plausible failure mode, supports physiological interpretation, or informs the next diagnostic action. Interpretability should therefore be evaluated by its contribution to error detection and clinical decisions rather than by visual plausibility alone. Promising approaches include morphology-rhythm decomposition, which can distinguish short-term morphological information from longer-term rhythm-related features (68). Useful criteria for clinically meaningful interpretability include reproducibility, perturbation stability, physiological plausibility, and clinician assessment (69). A visually appealing map alone is insufficient evidence of clinical interpretability. Explainability should not, however, be assumed to increase clinician trust automatically (70). Explanations that appear physiologically plausible but are poorly faithful to the underlying model may create false reassurance or encourage automation bias. Clinically useful explainability should instead promote calibrated trust: clinicians should be able to recognize when a prediction is physiologically plausible, when the model may be relying on an artifact or confounder, and when additional testing is warranted. The influence of explainability on adoption should therefore be evaluated in human-factor or prospective workflow studies using endpoints such as diagnostic accuracy, decision time, confidence calibration, disagreement handling, and inappropriate reliance on model output, rather than clinician preference for a visualization alone.

AI-ECG should generally remain under clinician oversight. The model may identify patterns that warrant attention, but the clinician must integrate symptoms, biomarkers, imaging, comorbidities, and pretest probability. This is especially important for structural heart disease and rare cardiomyopathies, where ECG abnormalities are neither fully sensitive nor disease-specific. This requirement is particularly important in high-stakes triage and rare-disease screening.

### 6.7 Ethical, equity, and accountability considerations

Ethical deployment of AI-ECG requires attention not only to privacy but also to bias, accountability, and the distribution of clinical benefit and harm. Algorithmic bias may arise when training data underrepresent specific demographic groups, disease phenotypes, devices, or care settings, or when labels reflect unequal access to confirmatory testing. Aggregate discrimination can therefore conceal clinically important subgroup failures (71). External and prospective studies should report performance and calibration across relevant demographic and clinical subgroups and investigate whether false-positive and false-negative burdens are distributed unequally.

Accountability is also important when AI-ECG influences screening, referral, or time-sensitive triage. Operational accountability should be explicitly defined across stakeholders involved in AI development, deployment, and clinical use, including model developers, healthcare institutions, and clinicians, particularly when proprietary systems provide limited information about training data, calibration, or known failure modes (72). AI outputs should therefore remain traceable, auditable, and subject to clinician oversight, with explicit protocols for disagreement between model recommendations and clinical judgment. Institutional governance should therefore specify who reviews AI-generated recommendations, how disagreements are resolved, when overrides are appropriate, and how these decisions are documented. Governance should aim for calibrated human–AI interaction rather than either autonomous decision-making or nominal oversight.

Finally, equitable implementation requires consideration of access. Systems that depend on specific digital infrastructure, proprietary ECG formats, or costly downstream testing may widen disparities even when model performance is technically similar across groups. Fairness should therefore be evaluated not only at the level of model performance but also across the AI lifecycle, including data representativeness, deployment processes, and downstream care pathways, where unequal access or follow-up capacity may amplify disparities (72, 73).

Resource-constrained settings may represent an important use case for AI-ECG because ECG acquisition is relatively inexpensive and portable compared with echocardiography or advanced cardiovascular imaging. However, low acquisition cost does not necessarily ensure feasible implementation. Limited specialist availability, heterogeneous low-cost devices, unstable connectivity, restricted access to confirmatory imaging or laboratory testing, and limited capacity for model maintenance and recalibration may reduce the clinical value of an otherwise accurate algorithm. Portable and point-of-care AI-ECG systems may extend screening or triage beyond specialist centers (36, 49), but implementation should be evaluated in relation to device robustness, infrastructure requirements, affordability, diagnostic yield, and the local availability of downstream confirmatory care.

### 6.8 Standardization, governance, and future priorities

Future priorities address three linked translational questions: whether reported performance is credible, whether it remains stable across deployment environments, and whether the system can be governed safely throughout its clinical lifecycle.

First, credible evidence requires standardized inputs and transparent study design. Standardized ECG formats and documented preprocessing may reduce avoidable variation arising from proprietary formats and inconsistent implementations (65). Transparent disease definitions and label-adjudication procedures clarify whether the prediction target matches the intended clinical use. Patient-level separation, prespecified thresholds, calibration, predictive values, and subgroup analyses should be treated as core indicators of evidentiary credibility. These practices determine whether reported performance represents disease-related information rather than leakage, label noise, or acquisition artifacts.

Second, transportability requires evaluation across institutions, devices, populations, and time. Standardization can reduce avoidable heterogeneity but cannot replace external validation. Federated learning may support multinational model development without centralizing institutional data, although external validation remains necessary to establish transportability (59, 74, 75). Participating centers may retain different disease definitions, devices, populations, and practice patterns. Federated models therefore require the same independent validation, calibration, and subgroup assessment as centrally trained systems.

Third, safe implementation requires prospective evaluation and lifecycle governance. ECG data may retain identifiable biometric information even after conventional metadata removal (76). Studies should embed AI-ECG into explicit care pathways and determine whether alerts change testing, referral, treatment, or time-sensitive decisions. Outcome-oriented evaluation should assess whether these changes benefit patients and whether harms are distributed equitably. Regulatory review, data protection, cybersecurity, audit trails, post-deployment monitoring, and procedures for updating models are necessary because performance may drift after changes in devices or clinical practice (77). Commercial systems should disclose intended use, validation populations, operating thresholds, known failure modes, and post-market performance whenever possible.

These priorities differ by clinical application. For OMI, the next critical step is a prospective interventional trial testing whether AI-guided catheterization-laboratory activation improves reperfusion decisions without unacceptable false activation. For prediction of occult or future AF, randomized studies should determine whether AI-guided prolonged monitoring increases clinically meaningful AF detection and ultimately reduces thromboembolic events without excessive monitoring burden. For VHD, HFpEF, and cardiac amyloidosis, prospective device-diverse studies should establish diagnostic yield, referral efficiency, and downstream resource use before broader clinical deployment is considered. Reduced-LVEF and HCM applications, which have already reached prospective implementation or controlled workflow evaluation, should now move toward treatment-related, patient-outcome, and cost-effectiveness endpoints. Across all applications, studies should prespecify operating thresholds, calibration procedures, subgroup analyses, ECG-quality failure criteria, and post-deployment drift monitoring.

### 6.9 Limitations of this review

This review has several limitations. First, AI-ECG research is developing rapidly, and the evidence base may change soon after publication. Second, the number and methodological quality of studies differ substantially across disease domains. This imbalance limits direct comparisons of translational maturity.

Third, studies report heterogeneous endpoints, thresholds, validation designs, and performance metrics. Quantitative comparison across applications should therefore be interpreted cautiously. Fourth, information on some commercial and real-world systems remains incomplete. Model architecture, training data, calibration, subgroup performance, and post-market experience may not be fully transparent.

As a critical narrative review, we did not perform a quantitative meta-analysis or a formal study-level risk-of-bias assessment. Substantial heterogeneity in clinical tasks, reference standards, validation designs, and reported metrics also precluded meaningful quantitative synthesis. These translational interpretations should therefore be viewed as a structured narrative synthesis rather than a quantitative ranking.

Publication bias may also influence the apparent maturity of AI-ECG applications. Studies reporting high discrimination, successful external validation, or favorable workflow effects may be more likely to be published than unsuccessful model-development efforts, negative external validations, or implementation studies showing no clinical benefit. This imbalance may be particularly relevant for commercial systems, for which negative performance data and post-deployment experience may be incompletely reported. Because gray literature, trial registries, and unpublished studies were not systematically searched, negative or neutral findings may also be underrepresented. Consequently, the evidence summarized here may overestimate both technical performance and translational readiness in some applications. In addition, study screening was not performed independently in duplicate, which may have increased the influence of reviewer judgment on study selection.

Finally, this review emphasizes intended clinical use, reference-standard reliability, external validation, actionability, and workflow evidence. This translational focus necessarily limits detailed coverage of every network architecture and algorithmic innovation. The review should therefore be interpreted as a critical assessment of the pathway from technical performance to clinical value, rather than an exhaustive catalog of AI-ECG methods.

## 7 Conclusion

This review provides a clinically oriented appraisal of translational maturity across AI-ECG applications. The available evidence indicates that clinical value depends less on peak discrimination than on the alignment of intended use, reference-standard validity, transportability, actionability, and demonstrated benefit within care pathways. AI-ECG has advanced furthest in STEMI reperfusion triage and screening for reduced left ventricular ejection fraction, where prospective or randomized evaluations have demonstrated workflow or targeted diagnostic benefits. AMI rule-out has reached prospective multicenter diagnostic validation but remains primarily supported by diagnostic-performance evidence rather than outcome-based implementation studies. Hypertrophic cardiomyopathy screening has also progressed through international validation and prospective implementation. OMI-oriented decision support is diagnostically promising but still lacks prospective interventional and patient-outcome evidence. Prediction of occult or future AF and applications in valvular heart disease, heart failure with preserved ejection fraction, long-term heart-failure risk, and cardiac amyloidosis remain primarily strategies for risk enrichment, suspicion raising, or referral support rather than stand-alone diagnosis.

Broader adoption therefore requires more than incremental gains in model performance. Future studies should use clinically aligned reference standards and patient-level data separation; report calibration, predictive values, prespecified thresholds, and subgroup performance; validate across institutions, populations, devices, and time; and embed model outputs in explicit pathways with clinician oversight and confirmatory testing. Prospective comparative studies should determine whether AI-ECG improves treatment decisions, patient outcomes, cost-effectiveness, and equity while monitoring privacy, cybersecurity, performance drift, and unintended harm. AI-ECG is thus best understood as an assistive layer that extends the information yield of a widely available test. Its durable contribution to cardiovascular care will depend on converting technically credible predictions into reproducible, safe, and outcome-relevant clinical action.
